# Identifying Patients at Risk for Cardiometabolic and Chronic Diseases by Using the Exercise Vital Sign to Screen for Physical Inactivity

**DOI:** 10.5888/pcd22.240149

**Published:** 2025-01-02

**Authors:** Cole G. Chapman, Mary C. Schroeder, Britt Marcussen, Lucas J. Carr

**Affiliations:** 1 Department of Pharmacy Practice and Science, University of Iowa, Iowa City; 2 Department of Family Medicine, University of Iowa Health Care, Iowa City; 3 Department of Health and Human Physiology, University of Iowa, Iowa City

## Abstract

**Introduction:**

Physical inactivity is a major health risk factor for multiple chronic diseases and early death. Despite evidence supporting diet and physical activity behavioral counseling interventions, physical inactivity is rarely measured or managed in primary care. A need exists to fully explore and demonstrate the value of screening patients for physical inactivity. This study aimed to 1) compare health profiles of patients screened for inactivity versus patients not screened for inactivity, and 2) compare health profiles of inactive, insufficiently active, and active patients as measured by the Exercise Vital Sign screener.

**Methods:**

The study sample comprised adult patients attending a well visit from November 1, 2017, through December 1, 2022, at a large midwestern university hospital. We extracted data from electronic medical records on exercise behavior reported by patients using the Exercise Vital Sign (EVS) questionnaire. We extracted data on demographics characteristics, resting pulse, encounters, and disease diagnoses from PCORnet Common Data Model (version 6.1). We used the Elixhauser Comorbidity Index to determine disease burden. We compared patients with complete and valid EVS values (n =7,261) with patients not screened for inactivity (n = 33,445). We conducted further comparisons between screened patients reporting 0 minutes (inactive), 1 to 149 minutes (insufficiently active), or ≥150 minutes (active) minutes per week of moderate-vigorous physical activity.

**Results:**

Patients screened for inactivity had significantly lower rates of several comorbid conditions, including obesity (*P* < .001), diabetes (*P* < .001), and hypertension (*P* < .001) when compared with unscreened patients. Compared with insufficiently active and inactive patients, active patients had a lower risk of 19 inactivity-related comorbid conditions including obesity (*P* < .001), depression (*P* < .001), hypertension (*P* < .001), diabetes (*P* < .001), and valvular disease (*P* < .001).

**Conclusion:**

These findings suggest inactive and insufficiently active patients are at increased risk for multiple inactivity-related chronic conditions. These findings further support existing recommendations that inactive patients receive or be referred to evidence-based lifestyle behavioral counseling programs.

**Summary**

**What is already known on this topic?**

Physical inactivity is a risk factor for multiple chronic diseases and early death. Despite evidence supporting diet and physical activity behavioral counseling interventions, physical inactivity is rarely measured or managed in primary care.

**What is added by this report?**

We used electronic medical records to link patient-reported physical activity to risk factors and disease diagnoses. Inactive patients are at higher risk for up to 19 inactivity-related comorbid conditions.

**What are the implications for public health practice?**

Treating physical activity as a vital sign and implementing physical inactivity screenings into primary care visits has value. Promoting physical activity among the least active and least healthy populations can reduce health disparities and improve population health.

**Instructions for Obtaining Continuing Education**

To receive continuing education (CE) for SCJA4894 — *Preventing Chronic Disease* Journal Series — please visit https://www.train.org/cdctrain/welcome and use **JA4894-122624** to search for the course in the Course Catalog. Follow the steps below by January 2, 2027.

1. Register for and complete the course.
2. Pass the post-assessment at 80%.
3. Complete the evaluation.
4. Visit Your Learning to access your certificates and transcript.

**Accreditation Statements**

In support of improving patient care, the Centers for Disease Control and Prevention is jointly accredited by the Accreditation Council for Continuing Medical Education (ACCME), the Accreditation Council for Pharmacy Education (ACPE), and the American Nurses Credentialing Center (ANCC), to provide continuing education for the healthcare team.

**Credit Hours**

**CME: **The Centers for Disease Control and Prevention designates this **enduring** activity for a maximum of **(1.0) **AMA PRA Category 1 Credits™. Physicians should claim only the credit commensurate with the extent of their participation in the activity.

**CNE: **The Centers for Disease Control and Prevention designates this activity for **(1.0)** nursing contact hours.

**CEU: **The Centers for Disease Control and Prevention is authorized by IACET to offer **(0.1)** CEUs for this program.

**CECH: **Sponsored by the Centers for Disease Control and Prevention, a designated provider of continuing education contact hours (CECH) in health education by the National Commission for Health Education Credentialing, Inc. This program is designated for Certified Health Education Specialists (CHES^®^) and/or Master Certified Health Education Specialists (MCHES^®^) to receive up to **(1.0) **total Category I continuing education contact hours. Maximum advanced level continuing education contact hours available are **(1.0)**. Continuing Competency credits available are** (1.0). **CDC provider number **98614.**

**CE origination date:** January 2, 2025; **CE expiration date**: January 2, 2027

**Program Description**

This enduring activity is designed to increase knowledge and change competency of public health practices and strategies.

**Objectives**

At the conclusion of the session, the participant should be able to:

1. Assess the effectiveness of existing public health programs and initiatives.
2. Explain how data can be used to improve program efficiency.
3. Describe the impact that the recommendations will make on public health.
4. Explain the appropriate time to use evidence in decision making.
5. Identify ways to enhance collaborative investments for strengthening evidence-based public health (EBPH) practice across the healthcare team.
6. Describe current public health information.

**Faculty/Credentials**

- Chapman, Cole, PhD, University of Iowa Health Care, Department of Pharmacy Practice and Science
- Schroeder, Mary, PhD, University of Iowa Health Care, Department of Pharmacy Practice and Science
- Marcussen, Britt, MD, University of Iowa Health Care, Department of Family Medicine
- Carr, Lucas, PhD, University of Iowa Health Care, Department of Health and Human Physiology

**Hardware/software:** Computer hardware; internet connection; browser

**Materials:** None

**Target audience:** Physicians, registered nurses, public health professionals, health educators

**Prerequisites:** None

**Format:** This activity is Journal

**Disclosure:** In compliance with continuing education requirements, all planners and presenters must disclose all financial relationships, in any amount, with ineligible companies during the previous 24 months as well as any use of unlabeled product(s) or products under investigational use. CDC, our planners, and content experts wish to disclose they have no financial relationship(s) with ineligible companies whose primary business is producing, marketing, selling, reselling, or distributing healthcare products used by or on patients. Content will not include any discussion of the unlabeled use of a product or a product under investigational use. CDC did not accept financial or in-kind support from ineligible companies for this continuing education activity.

**Fees:** No fees are charged for CDC’s CE activities.

## Introduction

Physical inactivity is a major risk factor for several leading causes of death including cardiovascular disease (CVD), cancer, respiratory disease, and diabetes (1). Early diagnosis and management of such chronic diseases in primary care can improve patient outcomes. Despite the well-documented benefits of physical activity for preventing and managing more than 25 chronic diseases and health conditions, including CVD, cancer, respiratory disease, and diabetes (2,3), patients are rarely screened for physical inactivity in primary care settings. This omission may contribute to the underdiagnosis and suboptimal management of chronic conditions related to inactivity (4–6).

In 2007, the American College of Sports Medicine launched the Exercise is Medicine initiative, which called for all health care providers to treat physical inactivity as a vital sign by screening patients for inactivity during all visits (7). According to this initiative, patients identified as insufficiently active should be prescribed exercise by their provider or referred to an evidence-based community resource to receive a physical activity program. The Exercise is Medicine recommendations directly align with 2 recent reports issued by the US Preventive Services Task Force (USPSTF). In 2020, the USPSTF issued a Grade B recommendation indicating that adults with at least 1 CVD risk factor (eg, dyslipidemia, elevated blood pressure, mixed risk factors) should receive or be referred to an evidence-based behavioral counseling intervention to improve diet and physical activity behaviors (8). This recommendation was based on supportive evidence indicating medium- and high-contact behavioral counseling interventions are effective in reducing CVD events and risk factors with little to no risk of serious harm (8). Furthermore, in 2022, the USPSTF issued a Grade C recommendation indicating that clinicians should individualize decisions to offer physical activity and dietary behavioral counseling interventions to healthy adults without CVD risk factors (9). Importantly, this recommendation indicates that clinical decisions for offering behavioral counseling interventions to people with CVD risk factors should be informed by the patient’s current level of physical activity or inactivity. Despite evidence supporting the effectiveness of physical activity behavioral counseling programs for improving patient outcomes (8), physical activity and inactivity are rarely measured or intervened upon in primary care.

The Exercise Vital Sign (EVS) and the Physical Activity Vital Sign (PAVS) are 2-item questionnaires that have been demonstrated to be quick and valid tools to identify patients who are not meeting the aerobic physical activity guidelines (10). To the best of our knowledge, 17 studies have examined the clinical utility of physical inactivity screening tools like the EVS or PAVS. Previous studies examined the relationship between patient’s physical activity and several indicators of health, including cardiometabolic risk factors (eg, body mass index [BMI], blood pressure, glucose levels, lipids, waist circumference, physical function), diagnosis of chronic conditions (eg, hypertension, dyslipidemia, hyperglycemia, metabolic syndrome, coronary artery disease, chronic obstructive pulmonary disease [COPD], COVID-19), mental health outcomes (eg, perceived mood, perceived anxiety, perceived stress, posttraumatic stress disorder [PTSD] symptom severity), and amputation characteristics (11–27). Ten of these studies used the PAVS and 7 studies used the EVS. In such studies, the most common health outcomes included were BMI (n = 9) and disease burden (n = 4). Most studies (n = 11) were conducted among patients from the US, with the remaining studies conducted in Belgium (n = 3), Sweden (n = 1), and Spain (n = 2). Five studies were conducted among generally healthy adult patients being treated in primary care, while others focused on specific clinical populations, including those diagnosed with COVID-19, schizophrenia, bipolar disorder, COPD, major amputations, and PTSD.

To date, few studies have leveraged the full power of electronic medical record (EMR) data by examining the wholistic relationship between patients’ clinically screened physical inactivity and all health outcomes commonly captured in the EMR. Additionally, only 2 studies compared health outcomes among patients who had been screened for inactivity versus patients who had not been screened, which limits our understanding of whether screened patients are representative of all patients being treated in a given hospital system. Finally, while BMI is the most common health outcome examined in studies exploring the use of the EVS and PAVS, no studies have been conducted among patients residing in the midwestern region of the US, where prevalence of obesity is especially high (28).

Collectively, a need exists for more evidence on the uses and potential value of screening patients for physical inactivity. Research in this area could inform clinical decisions that have potential to improve patient outcomes. To advance this work, this study aimed to 1) compare health profiles of patients screened for inactivity versus patients not screened for inactivity and 2) compare health profiles of patients identified as inactive, insufficiently active, and active as measured by the EVS. We hypothesized that patients screened for inactivity and patients not screening for inactivity during annual wellness visits would have similar cardiometabolic health profiles and a similar disease burden. We also hypothesized that active patients would have healthier cardiometabolic profiles and a lower disease burden than patients who reported insufficient levels of physical activity.

## Methods

### Data collection and cohort

Data for this study came from the EMRs of adult patients (aged ≥18 y) seen during an annual wellness visit in a family medicine clinic at a large, public university hospital system in the Midwest. The study sample included 2 distinct cohorts, each identified from EMRs for the period from November 1, 2017, through December 1, 2022. The first cohort (the EVS cohort) included all patients screened for physical inactivity with the EVS during a general physical examination, an annual wellness examination, or a Welcome to Medicare visit. The second cohort (the comparison cohort) included all patients who had a visit of the same type and in a department represented in the first sample, but these patients were not screened for inactivity during their visit. We conducted this comparison to ascertain whether the small fraction of patients who were screened for inactivity were similar to the overall patient population seen at this hospital during the study period. We excluded patients with any indication of an EVS record from the comparison cohort, regardless of whether the EVS record was populated with complete or valid information. We excluded patients in the EVS cohort if EVS data elements were missing or invalid on their index visit, defined as the first encounter for a visit with EVS data (EVS cohort) or a type of encounter in which an EVS measurement was indicated (comparison cohort). We considered EVS data invalid if days per week and minutes per day did not match; for example, if the patient indicated they had exercised 20 minutes per day for 0 days per week.

The data extract conformed to the PCORnet Common Data Model version 6.1 (PCORnet Network Partners). Data elements included patient characteristics and their encounters with the health care system during the study period. For analyses, we aggregated data to the patient level. Each observation of the analysis dataset characterized the patient at the time of the index visit and the 365-day period before and after the index visit. Finally, after review by the Human Subjects Office at the University of Iowa, it was determined this study did not meet the regulatory definition of human subjects research and thus did not require institutional review board approval.

### Measures

#### Exercise Vital Sign (EVS)

Physical inactivity was screened by using the 2-item EVS. Patients completed the questionnaire on a tablet provided to them during the rooming process. The EVS asks 2 questions: “On average, how many days per week do you engage in moderate to vigorous exercise (like a brisk walk)?” (0–7 days) and “On average, how many minutes do you engage in exercise at this level?” Each EVS record obtained from the EMR consisted of 2 component values associated with a single patient encounter. The component values were 1) the patient-reported average minutes of activity per day and 2) the patient-reported average days per week of activity. The total minutes of activity per week was calculated by multiplying the 2 component EVS values. We then created a categorical variable, activity level, from the minutes-per-week measure. We defined 3 groups of activity level: inactive (0 minutes per week), insufficiently active (1–149 minutes), and active (>149 minutes). When multiple nonmissing values of an EVS component were associated with a single encounter, we used the average value. 

#### Patient and visit characteristics

Measures created for this analysis included patient demographic characteristics, the number and types of comorbid conditions, resting pulse, laboratory values, and smoking status. Demographics characteristics were age at the time of the index visit, race, ethnicity, and sex (all self-reported by the patient). We combined categories of race and ethnicity that represented less than 2% of the patient population into the category “Other/unknown.” We used the Elixhauser Comorbidity Index (29) to assess the number and type of comorbid conditions specified in clinical and billing diagnosis codes recorded in the 365 days before the index visit. The Elixhauser Comorbidity Index identifies and summarizes 38 pre-existing conditions. We created a binary variable for each Elixhauser condition to indicate whether any relevant diagnosis code was present in the medical record. We excluded from analysis binary variables (n = 14) that had a frequency of less than 2% of the overall sample size, resulting in 24 conditions. We created an additional single variable to measure the sum of the number of Elixhauser pre-existing conditions observed.

We collected data on the following: resting pulse, systolic blood pressure, diastolic blood pressure, height, weight, and BMI. Laboratory values, when available, were hemoglobin A_1c_, and cholesterol (high-density lipoprotein [HDL], low-density lipoprotein [LDL]), and triglycerides. We estimated all values as median values across all available values observed within 365 days before or after the index visit (a 730-day window), based on the visit date and specimen date, respectively.

Data on smoking status, self-reported by patients, were available in the EMR. When multiple values were reported, a value indicating positive smoking status took priority over a negative status (ie, current smoker overrode former smoker, and former smoker overrode never smoker). We filled any missing values with values from other encounters. Any known status was assumed to be stable until a new status was observed.

### Statistical analyses

We tested differences in clinical and demographic characteristics across the EVS and comparison cohorts by using the Welch 2-sample *t* test for continuous variables and the test of equal proportions for binary variables. We used logistic regression to estimate and test the independent associations between measured variables and having any EVS value (ie, EVS cohort vs comparison cohort). When more than 2% of the overall sample population was missing data for laboratory values (eg, resting heart rate, blood pressure, BMI), we excluded these from the set of covariables included as independent variables in the logistic regression; we also excluded diagnosis-based conditions present among less than 2% of the overall sample population. We then compared median values of clinical and demographic measures across 3 levels of reported physical activity among the subset of patients with a valid EVS. We performed a linear test of trend for each measure across the 3 activity levels: inactive, insufficiently active, and active. We then used logistic regression to estimate and test independent associations of these variables with activity level in the EVS cohort. The dependent variable in this model was the active category (ie, active vs combined inactive or insufficiently active). The set of independent variables was consistent with the set used in the model predicting any EVS value. We used R version 4.3.1 (R Foundation for Statistical Computing) to conduct all analyses.

## Results

We identified 7,261 patients in the EVS cohort and 33,444 patients in the comparison cohort during the study period (Table 1). Patients in the EVS cohort were slightly younger (median age 40 y) than those in the comparison cohort (median age 42 y). The EVS cohort had significantly lower values than the comparison cohort for BMI, systolic blood pressure, diastolic blood pressure, resting pulse, triglycerides, and HbA_1c_. However, these differences were generally small and not clinically significant.

**Table 1:** Demographic Characteristics and Health Outcomes Among Patients Screened and Not Screened With the Exercise Vital Sign (EVS) and Results of Logistic Regression Model Predicting Screening With EVS at a Large Midwestern University Hospital, 2017–2022

Characteristic	Cohort summary^a^				Logistic regression estimates, odds ratio (95% CI) [*P* value]
	Overall (N = 40,705)	Comparison cohort (n = 33,444)	EVS cohort (n = 7,261)	*P* value^b^	
**Age at index, y**	42 (29–58)	42 (30–58)	40 (27–57)	<.001	1.00 (0.99–1.00) [<.001]
**Sex^c^**					
Female	22,710 (56)	18,739 (56)	3,971 (55)	.04	1 [Reference]
Male	17,992 (44)	14,703 (44)	3,289 (45)	.04	1.15 (1.09–1.22) [<.001]
**Race and ethnicity**					
Asian	2,184 (5.4)	1,493 (4.5)	691 (9.5)	<.001	2.13 (1.93–2.35) [<.001]
Black or African American	3,180 (7.8)	2,561 (7.7)	619 (8.5)	.01	1.23 (1.12–1.36) [<.001]
Hispanic or Latino of any race	2,325 (5.7)	2,023 (6.0)	302 (4.2)	<.001	0.70 (0.61–0.79) [<.001]
White	31,429 (77.2)	26,075 (78.0)	5,354 (73.7)	<.001	1 [Reference]
Multiracial	832 (2.0)	678 (2.0)	154 (2.1)	.64	1.01 (0.84–1.21) [.87]
Other or unknown	755 (1.9)	614 (1.8)	141 (1.9)	.58	1.10 (0.91–1.33) [.31]
**Ethnicity**					
Hispanic	2,754 (6.8)	2,380 (7.1)	374 (5.2)	<.001	—d
Not Hispanic	37,342 (91.7)	30,568 (91.4)	6,774 (93.3)	<.001	—d
Other or unknown	609 (1.5)	496 (1.5)	113 (1.6)	.68	—d
**Smoking status**					
Current	5 (<0.1)	5 (<0.1)	0	.65	—e
Former	7,061 (17.3)	5,917 (17.7)	1,144 (15.8)	<.001	—e
Never	15,054 (37.0)	12,078 (36.1)	2,976 (41.0)	<.001	—e
Unknown	18,585 (45.7)	15,444 (46.2)	3,141 (43.3)	<.001	—e
**Body mass index**	28 (24–33)	28 (24–34)	27 (23–31)	<.001	—e
**Blood pressure, mm Hg**					
Systolic	125 (117–134)	125 (117–134)	124 (115–133)	<.001	1.02 (1.02–1.03) [<.001]
Diastolic	77 (72–82)	77 (72–83)	75 (69–80)	<.001	0.94 (0.93–0.94) [<.001]
**Resting pulse, beats per min**	78 (70–85)	78 (71–86)	76 (69–84)	<.001	—e
**Lipids, mg/dL**					
High-density lipoprotein cholesterol	51 (42–63)	51 (42–62)	53 (43–65)	<.001	—e
Low-density lipoprotein cholesterol	99 (78–123)	99 (78–123)	99 (78–123)	.92	—e
Triglycerides	110 (75–163)	112 (76–166)	103 (71–151)	<.001	—e
**Hemoglobin A_1c_, %**	5.50 (5.20–5.90)	5.50 (5.20–5.90)	5.40 (5.10–5.70)	<.001	—e
**Elixhauser Comorbidity Index**	1.0 (0–2.0)	1.0 (0–2.0)	1.0 (0–2.0)	<.001	0.95 (0.89–1.00) [.06]
**Clinical conditions**					
Anemia deficiency	2,381 (5.8)	2,044 (6.1)	337 (4.6)	<.001	0.80 (0.69–0.93) [.004]
Autoimmune disorder	882 (2.2)	720 (2.2)	162 (2.2)	.71	1.18 (0.97–1.41) [.09]
Cancer solid tumor	1,335 (3.3)	1,107 (3.3)	228 (3.1)	.48	1.08 (0.91–1.27) [.38]
Cerebrovascular disease	669 (1.6)	545 (1.6)	124 (1.7)	.67	—f
Chronic pulmonary disease	4,583 (11.3)	3,799 (11.6)	784 (10.8)	.18	1.08 (0.97–1.20) [.16]
Congestive heart failure	720 (1.8)	643 (1.9)	77 (1.1)	<.001	—f
Depression	7,607 (18.7)	6,364 (19.0)	1,243 (17.1)	<.001	1.01 (0.92–1.12) [.79]
Diabetes complicated	2,742 (6.7)	2,426 (7.3)	316 (4.4)	<.001	0.72 (0.61–0.86) [<.001]
Diabetes uncomplicated	3,276 (8.0)	2,849 (8.5)	427 (5.9)	<.001	0.93 (0.80–1.08) [.32]
Drug abuse	607 (1.5)	496 (1.5)	111 (1.5)	.81	—f
Hypertension uncomplicated	10,797 (26.5)	9,233 (27.6)	1,564 (21.5)	<.001	0.93 (0.84–1.02) [.13]
Hypertension complicated	1,006 (2.5)	850 (2.5)	156 (2.1)	.06	1.26 (1.01–1.57) [.04]
Hypothyroidism	4,168 (10.2)	3,496 (10.5)	672 (9.3)	.002	1.04 (0.93–1.16) [.52]
Liver disease mild	1,315 (3.2)	1,140 (3.4)	175 (2.4)	<.001	0.89 (0.74–1.06) [.20]
Neurologic disorders affecting movement	837 (2.1)	721 (2.2)	116 (1.6)	.003	0.89 (0.72–1.10) [.28]
Neurologic disorders other	584 (1.4)	488 (1.5)	96 (1.3)	.40	—f
Neurologic seizures	723 (1.8)	615 (1.8)	108 (1.5)	.04	—f
Obesity	7,131 (17.5)	6,062 (18.1)	1,069 (14.7)	<.001	1.01 (0.92–1.12) [.78]
Peripheral vascular disease	1,097 (2.7)	928 (2.8)	169 (2.3)	.04	1.00 (0.83–1.21) [.98]
Psychoses	2,679 (6.6)	2,174 (6.5)	505 (7.0)	.16	1.31 (1.16–1.49) [<.001]
Renal failure moderate	984 (2.4)	852 (2.5)	132 (1.8)	<.001	0.88 (0.70–1.09) [.25]
Thyroid other	1,212 (3.0)	1,014 (3.0)	198 (2.7)	.18	1.03 (0.86–1.21) [.76]
Valvular disease	690 (1.7)	576 (1.7)	114 (1.6)	.39	1.04 (0.82–1.29) [.76]
Weight loss	885 (2.2)	737 (2.2)	148 (2.0)	.41	1.01 (0.82–1.23) [.93]

Abbreviation: —, does not apply.

a Values are median (IQR) for continuous measures and number (percentage) for categorical measures. The EVS cohort included all patients screened for physical inactivity with the EVS during a general physical examination, an annual wellness examination, or a Welcome to Medicare visit. Patients in the comparison cohort were not screened for physical inactivity.

b Welch 2-sample *t* test for continuous measures; 2-sample test for equality of proportions without continuity correction for categorical measures.

c Not all participants answered this question.

d Demographic variable excluded from model due to strong correlation with the race variable.

e Vital sign or laboratory variable excluded from the model due to a high frequency of missing values.

f Diagnosis variable excluded due to low frequency.

The comorbidity burden, as determined by the Elixhauser Comorbidity Index, was similar in the EVS cohort and the comparison cohort. However, the EVS cohort had significantly lower rates of several comorbid conditions, including obesity (15% vs 18%), depression (17% vs 19%), hypertension (22% vs 28%), uncomplicated diabetes (5.9% vs 8.5%), complicated diabetes (4.4% vs 7.3%), anemia deficiency (4.6% vs 6.1%), hypothyroidism (9.3% vs 10.0%), congestive heart failure (1.1% vs 1.9%), and moderate renal failure (1.8% vs 2.5%). We observed no other significant differences in comorbid conditions between the EVS cohort and comparison cohort. 

Patients who self-reported Black/African American or Asian race had significantly greater odds of being in the EVS cohort than patients who self-reported White race (OR = 1.23 and 2.13, respectively) (Table 1). Patients who self-reported being Hispanic or Latino of any race had significantly lower odds of being in the EVS cohort than patients who self-reported White race (OR = 0.70). Presence of chronic conditions was not generally or consistently associated with being in the EVS cohort. Clinical conditions with significant odds ratio estimates were psychoses (OR = 1.31), complicated diabetes (OR = 0.72), complicated hypertension (OR = 1.26), and anemia deficiency (OR = 0.80).

### Physical activity level

Of the 7,261 patients in the EVS cohort, 4,382 (60%) patients were considered active, 2,607 (36%) were insufficiently active, and 272 (4%) were inactive. Insufficiently active patients most commonly reported engaging in 2 or 3 days of activity per week and 30 minutes of activity per day. Active patients most commonly reported engaging in 5 days of activity per week and 60 minutes of activity per day (Figure).

**Figure:**
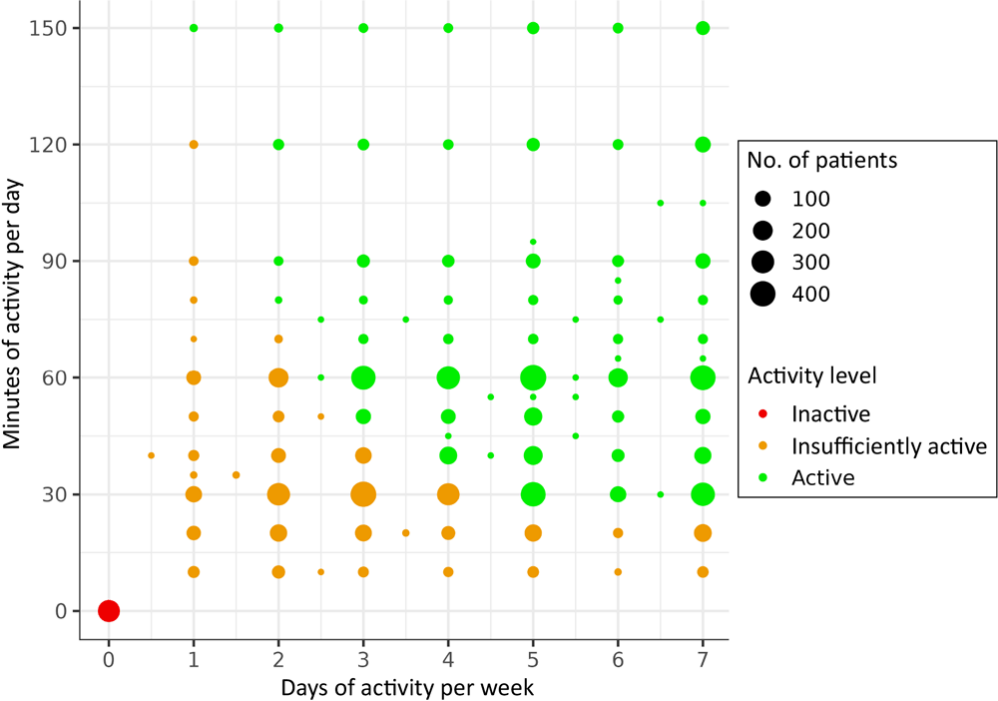
Distribution of activity level among 7,261 patients who were screened with the Exercise Vital Sign at a at a large midwestern university hospital, 2017–2022.

Compared with inactive and insufficiently active patients, active patients had significantly lower diastolic blood pressure, resting pulse, LDL cholesterol, and triglycerides, and significantly higher HDL cholesterol (Table 2). HbA_1c_ was significantly lower among active patients, but the difference was not clinically meaningful. We observed a significant trend in the proportion of former smokers, such that 29% of inactive patients reporting being a former smoker, compared with 15% of insufficiently active patients and 15% of active patients.

**Table 2:** Demographic Characteristics and Health Outcomes, by Physical Activity Level, Among Patients Screened With the Exercise Vital Sign (EVS) and Results of Logistic Regression Model Predicting an Active Activity Level Among EVS-Screened Patients at a Large Midwestern University Hospital, 2017–2022

Characteristic	EVS cohort^a^ (n = 7,261)				Logistic regression estimates, odds ratio (95% CI) [*P* value]
	Active (n = 4,382)	Insufficiently active (n = 2,607)	Inactive (n = 272)	*P* value^b^	
**Date of index visit**	November 2018–September 2022	November 2018–September 2022	June 2021–September 2022	**—**	**—**
**EVS measures**					
No. of active days per week	5.0 (4.0–7.0)	3.0 (2.0–4.0)	0 (0–0)	<.001	—
No. of active minutes per day	60 (40–60)	30 (20–30)	0 (0–0)	<.001	—
No. of active minutes per week	250 (180–360)	90 (60–120)	0 (0–0)	<.001	—
**Age at index, y**	41 (26–58)	40 (28–55)	39 (27–59)	.69	1.00 (1.00–1.01) [.34]
**Sex^c^**					
Female	2,245 (51)	1,568 (60.1)	158 (58.1)	<.001	1 [Reference]
Male	2,136 (49)	1,039 (39.9)	114 (41.9)	<.001	1.40 (1.26–1.55) [<.001]
**Race**					
Asian	386 (8.8)	281 (10.8)	24 (8.8)	.02	0.70 (0.59–0.83) [<.001]
Black or African American	311 (7.1)	260 (10.0)	48 (18)	<.001	0.64 (0.54–0.77) [<.001]
Hispanic/Latino	179 (4.1)	106 (4.1)	17 (6.3)	.21	0.89 (0.70–1.14) [.36]
White	3,323 (76)	1,871 (71.8)	160 (58.8)	<.001	1 [Reference]
Multiracial	102 (2.3)	41 (1.6)	11 (4.0)	.009	1.19 (0.84–1.69) [.34]
Other/unknown	81 (1.8)	48 (1.8)	12 (4.4)	.01	0.77 (0.54–1.08) [.13]
**Ethnicity**					
Hispanic	225 (5.1)	129 (4.9)	20 (7.4)	.23	—d
Not Hispanic	4,090 (93.3)	2,441 (93.6)	243 (89.3)	.03	—d
Other/unknown	67 (1.5)	37 (1.4)	9 (3.3)	.06	—d
**Smoking status**					
Current	0	0	0		—e
Former	673 (15.4)	391 (15.0)	80 (29.4)	<.001	—e
Never	1,795 (41.0)	1,134 (43.5)	47 (17.3)	<.001	—e
Unknown	1,914 (43.7)	1,082 (41.5)	145 (53.3)	<.001	—e
**Body mass index**	26 (23–30)	27 (24–33)	28 (24–33)	.58	—e
**Blood pressure, mm Hg**					
Systolic	124 (116–133)	124 (115–133)	124 (117–135)	.053	1.01 (1.00–1.02) [.001]
Diastolic	74 (69–80)	75 (70–81)	76 (71–81)	.002	0.98 (0.97–0.99) [<.001]
**Resting pulse, beats per min**	75 (68–83)	78 (71–86)	80 (73–88)	<.001	—e
**Lipids, mg/DL**					
High-density lipoprotein cholesterol	54 (44–66)	51 (42–62)	46 (39–57)	<.001	—e
Low-density lipoprotein cholesterol	99 (78–123)	100 (79–124)	102 (79–125)	.68	—e
Triglycerides	97 (67–142)	113 (77–164)	117 (86–182)	<.001	—e
**Hemoglobin A_1c_**	5.40 (5.10–5.70)	5.40 (5.10–5.80)	5.60 (5.30–6.11)	<.001	—e
**Elixhauser Comorbidity Index**	1.0 (0–2.0)	1.0 (0–2.0)	1.0 (0–3.0)	<.001	1.02 (0.91–1.14) [.75]
**Chronic conditions**					
Anemia deficiency	170 (3.9)	134 (5.1)	33 (12.1)	<.001	0.87 (0.66–1.14) [.31]
Autoimmune disorder	82 (1.9)	75 (2.9)	5 (1.8)	.02	0.69 (0.49–0.98) [.04]
Cancer solid tumor	144 (3.3)	73 (2.8)	11 (4.0)	.36	1.15 (0.84–1.58) [.40]
Cerebrovascular disease	83 (1.9)	36 (1.4)	5 (1.8)	.27	—f
Chronic pulmonary disease	438 (10.0)	304 (11.7)	42 (15.4)	.004	0.92 (0.75–1.11) [.37]
Congestive heart failure	43 (1.0)	26 (1.0)	8 (2.9)	.009	—f
Depression	648 (14.8)	523 (20.1)	72 (26.5)	<.001	0.75 (0.62–0.90) [.002]
Diabetes complicated	146 (3.3)	139 (5.3)	31 (11.)	<.001	0.64 (0.46–0.91) [.01]
Diabetes uncomplicated	214 (4.9)	184 (7.1)	29 (11.4)	<.001	0.95 (0.71–1.29) [.75]
Drug abuse	60 (1.4)	42 (1.6)	9 (3.3)	.04	—f
Hypertension complicated	78 (1.8)	67 (2.6)	11 (4.0)	.008	0.96 (0.64–1.46) [.86]
Hypertension uncomplicated	880 (20.1)	588 (22.6)	96 (35.3)	<.001	0.88 (0.73–1.07) [.19]
Hypothyroidism	386 (8.8)	269 (10.3)	17 (6.3)	.02	1.00 (0.81–1.23) [.99]
Liver disease mild	87 (2.0)	77 (3.0)	11 (4.0)	.008	0.77 (0.55–1.08) [.13]
Neurologic disorders affecting movement	58 (1.3)	49 (1.9)	9 (3.3)	.01	0.77 (0.51–1.15) [.20]
Neurologic disorders other	51 (1.2)	41 (1.6)	4 (1.5)	.34	—f
Neurologic seizures	53 (1.2)	46 (1.8)	9 (3.3)	.007	—f
Obesity	531 (12.1)	481 (18.5)	57 (21.0)	<.001	0.68 (0.57–0.81) [<.001]
Peripheral vascular disease	86 (2.0)	72 (2.8)	11 (4.0)	.02	0.70 (0.49–0.99) [.04]
Psychoses	266 (6.1)	202 (7.7)	37 (14.)	<.001	0.85 (0.67–1.08) [.19]
Renal failure moderate	73 (1.7)	53 (2.0)	6 (2.2)	.48	1.09 (0.71–1.69) [.70]
Thyroid other	115 (2.6)	76 (2.9)	7 (2.6)	.76	1.02 (0.75–1.40) [.89]
Valvular disease	56 (1.3)	47 (1.8)	11 (4.0)	<.001	0.65 (0.43–0.98) [.04]
Weight loss	79 (1.8)	57 (2.2)	12 (4.4)	.01	0.87 (0.61–1.26) [.46]

Abbreviation: —, does not apply.

a Values are median (IQR) for continuous measures and number (percentage) for categorical measures.

b Test for linear trend (*F* statistic).

c Not all participants answered this question.

d Demographic variable excluded from model due to strong correlation with the race variable.

e Vital sign or laboratory variable excluded from the model due to a high frequency of missing values.

f Diagnosis variable excluded due to low frequency.

The mean number of Elixhauser comorbid conditions had a significant downward trend as activity level increased: inactive patients had a mean of 2.2 conditions, insufficiently active patients had a mean of 1.5 conditions, and active patients had a mean of 1.2 conditions. However, the estimated odds ratio (OR = 1.02; 95% CI, 0.91–1.14; *P* = .75) for the median Elixhauser Comorbidity Index predicting active status was not significant (Table 2).

Compared with inactive and insufficiently active patients, active patients had significantly lower rates of up to 19 inactivity-related comorbid conditions, including obesity, mild liver disease, psychoses, chronic pulmonary disease, depression, weight loss, uncomplicated and complicated hypertension, uncomplicated and complicated diabetes, anemia deficiency, peripheral vascular disease, neurologic disorders affecting movement, neurologic seizures, autoimmune disorders, hypothyroidism, valvular disease, congestive heart failure, and drug abuse (Table 2). All significant odds ratio estimates for the set of clinical condition variables were less than 1, indicating that additional disease is associated with less physical activity as measured by the EVS.

## Discussion

The findings of our study suggest that patients who were screened for physical inactivity with the EVS were slightly younger and had lower levels of several CVD risk factors than unscreened patients. Few studies have compared the health outcomes of patients screened for inactivity versus unscreened patients, and none, to our knowledge, have comprehensively analyzed multiple health outcomes that are commonly available in EMRs. That EVS-screened patients are healthier than unscreened patients contradicts our hypothesis and is inconsistent with previous findings by Coleman and colleagues (12). In a study of 696,267 Kaiser Permanente patients in California, patients who were screened for inactivity with the EVS were found to be slightly older and had more comorbid conditions than patients who were not screened with the EVS. A major difference between our study and the Kaiser Permanente study is that Kaiser Permanente Southern California screens nearly all outpatients (86%) with the EVS whereas our institution limits screenings to patients attending annual wellness visits in one family medicine clinic. In our cohort of EVS-screened patients, 60.3% reported meeting the moderate-intensity aerobic physical activity guidelines (2), which is double the percentage of Kaiser Permanente patients (30.4%) who reported being sufficiently active. These findings suggest that patients attending annual wellness visits and included in this study were more active and healthier than the typical patient being treated in our health care system and likely reflect “the worried well.” The Exercise is Medicine initiative recommends that health care systems treat physical inactivity as a vital sign by screening all patients for inactivity during all visits (30). At a minimum, inactivity screenings should be implemented in specialty clinics that treat patients for inactivity-related conditions such as CVD, obesity, diabetes, and cancer. Limiting inactivity screenings to specific clinical populations or specific clinical visits decreases the number of opportunities for early intervention or prevention of inactivity-related diseases.

Of the patients who were screened for inactivity in our study, active patients had significantly healthier cardiometabolic profiles (6 healthier cardiometabolic risk factors) and lower risk of up to 19 inactivity-related comorbid conditions compared with inactive and insufficiently active patients. These findings support our hypothesis and are mostly consistent with previous studies. For example, similar to the Kaiser Permanente study (12), we found active patients screened with the EVS had a lower BMI and a lower burden of chronic disease than insufficiently active patients. Similar to the findings of Young et al (13), who examined relationships between EVS and cardiometabolic risk factors of 622,897 Kaiser Permanente Southern California patients, we found active patients had lower diastolic blood pressure and better glucose control (HbA_1c_) than insufficiently active patients.

Our findings can also be compared with studies that used the PAVS screening tool, which asks about general physical activity as opposed to exercise-specific activity (18,23). Our findings are consistent with the findings of Ball and colleagues, who observed that inactive patients being treated at Intermountain Healthcare in Utah (N = 34,712) had a higher BMI and higher burden of chronic disease compared with sufficiently active patients (18). Finally, our findings are consistent with the findings of McCarthy et al (23), who observed that inactive adults seeking care in a preventive cardiology clinic in New York (N = 951) had higher BMI and higher triglycerides when compared with active adult patients.

### Strengths and limitations

This study has strengths and limitations. First, our study adds to the few studies that have compared health outcomes among patients who have been screened for inactivity versus patients who have not. However, given our observation that EVS-screened patients were generally younger and healthier than unscreened patients, our findings on the relationship between physical activity and measured health outcomes should be interpreted with caution. The low prevalence of inactivity observed among patients screened for inactivity suggests that this sample is not representative of the typical patient population. Still, if the less healthy unscreened cohort had been screened for inactivity, it is likely that the observed relationship between physical inactivity and these health outcomes would be even stronger. Second, our study is among the first to report on the wholistic relationship between patients’ physical activity level and their cardiometabolic risk and disease burden. Past studies focused on a few selected risk factors, such as BMI and resting blood pressure, but they did not report on the many health outcomes known to improve with regular physical activity. We intentionally reported these findings to illustrate the well-known and wide-ranging health benefits of regular physical activity (2,3) and the value of treating physical inactivity as a vital sign among all patients (7,30). To our knowledge, ours is the first study to report data on a physical activity vital sign among adult patients residing in the Midwest, which is noteworthy because nearly 69 million people (20.6% of US population) reside in the Midwest (31) and midwestern states have higher-than-normal prevalence rates of physical inactivity (25.2%) (32) and obesity (35.8%) (28). However, given that social desirability bias may affect self-reported measures of physical activity and that we relied on a physical inactivity screening conducted at a single point in time, our results should be interpreted with caution.

### Conclusion

Despite the known health benefits of engaging in regular physical activity, patients are rarely screened for physical inactivity in health care. We explored the utility of a physical inactivity screener (the Exercise Vital Sign) for identifying patients at risk for inactivity-related risk factors and disease diagnoses. Inactive patients are at higher risk for up to 19 inactivity-related comorbid conditions. These findings support calls to treat physical activity as a vital sign by regularly screening patients for inactivity and providing inactive patients with resources to promote physical activity. 
